# Supplementary material for: High-throughput interrogation of programmed ribosomal frameshifting in human cells
Source: Nat Commun. 2020 Jun 16;11:3061. doi: 10.1038/s41467-020-16961-8 (PMC7297798; doi:10.1038/s41467-020-16961-8)
Supplement: Supplementary file 3 — Reporting Summary [file 41467_2020_16961_MOESM3_ESM.pdf]

nature research

Corresponding author(s): Eran Segal, Martin Mikl

Last updated by author(s): May 13, 2020

# Reporting Summary

Nature Research wishes to improve the reproducibility of the work that we publish. This form provides structure for consistency and transparency in reporting. For further information on Nature Research policies, see Authors & Referees and the Editorial Policy Checklist.

## Statistics

For all statistical analyses, confirm that the following items are present in the figure legend, table legend, main text, or Methods section.

| n/a | Confirmed | |
|---|---|---|
| ☐ | ☒ | The exact sample size (*n*) for each experimental group/condition, given as a discrete number and unit of measurement |
| ☐ | ☒ | A statement on whether measurements were taken from distinct samples or whether the same sample was measured repeatedly |
| ☐ | ☒ | The statistical test(s) used AND whether they are one- or two-sided<br>*Only common tests should be described solely by name; describe more complex techniques in the Methods section.* |
| ☐ | ☒ | A description of all covariates tested |
| ☐ | ☒ | A description of any assumptions or corrections, such as tests of normality and adjustment for multiple comparisons |
| ☐ | ☒ | A full description of the statistical parameters including central tendency (e.g. means) or other basic estimates (e.g. regression coefficient) AND variation (e.g. standard deviation) or associated estimates of uncertainty (e.g. confidence intervals) |
| ☐ | ☒ | For null hypothesis testing, the test statistic (e.g. *F*, *t*, *r*) with confidence intervals, effect sizes, degrees of freedom and *P* value noted<br>*Give P values as exact values whenever suitable.* |
| ☒ | ☐ | For Bayesian analysis, information on the choice of priors and Markov chain Monte Carlo settings |
| ☒ | ☐ | For hierarchical and complex designs, identification of the appropriate level for tests and full reporting of outcomes |
| ☐ | ☒ | Estimates of effect sizes (e.g. Cohen's *d*, Pearson's *r*), indicating how they were calculated |

*Our web collection on statistics for biologists contains articles on many of the points above.*

## Software and code

Policy information about availability of computer code

| | |
|---|---|
| Data collection | BD FACSDiva, Illumina NextSeq Control Software |
| Data analysis | Python 2.7.11 with pandas 0.20.3, numpy 1.13.1, seaborn 0.6, scipy 0.17, scikit-learn 0.18.2 and shap 0.28.5; custom-made scripts provided (https://github.com/martinmikl/PRF_mpra) |

For manuscripts utilizing custom algorithms or software that are central to the research but not yet described in published literature, software must be made available to editors/reviewers. We strongly encourage code deposition in a community repository (e.g. GitHub). See the Nature Research guidelines for submitting code & software for further information.

## Data

Policy information about availability of data

All manuscripts must include a data availability statement. This statement should provide the following information, where applicable:

- Accession codes, unique identifiers, or web links for publicly available datasets
- A list of figures that have associated raw data
- A description of any restrictions on data availability

The raw NGS data has been deposited at the NCBI GEO (GSE145684).
Information on PRF sites in viral, bacterial and eukaryotic genomes was gathered from FSDB (http://wilab.inha.ac.kr/fsdb/) and the cited literature.
HIV sequences have been retrieved from the HIV genome database (http://www.hiv.lanl.gov/).

# Field-specific reporting

Please select the one below that is the best fit for your research. If you are not sure, read the appropriate sections before making your selection.

☒ Life sciences ☐ Behavioural & social sciences ☐ Ecological, evolutionary & environmental sciences

For a reference copy of the document with all sections, see nature.com/documents/nr-reporting-summary-flat.pdf

# Life sciences study design

All studies must disclose on these points even when the disclosure is negative.

| | |
|---|---|
| Sample size | Choosing sample sizes in the design of sequence libraries constitutes a trade-off between library complexity and statistical power. Typically, every sequence manipulation (corresponding to a biological hypothesis) was tested in up to 15 sequence contexts, roughly corresponding with the sample size. This number was based on the number of PRF sites reported in the literature that were used for systematic sequence manipulations. |
| Data exclusions | Data was excluded solely based on technical problems (i.e. not enough reads (<threshold) mapping to a specific variants) as described in the Methods. We applied a number of filters to the raw sequencing data to reduce experimental noise. First, variants with less than 20 reads (perfect matches along the whole length) mapped across bins were removed. Second, for bins with a read count of less than three, the bin value was set to zero. Third, for each variant we set to zero bins surrounded by zero values as these constituted isolated bins unlikely to come from the actual distribution. Forth, to reduce bias coming from the open bins at the extreme values, we set their count to match their neighbor's if it was higher, as these bins are defined as containing the tails of the distribution of variants with no GFP fluorescence and maximal GFP expression, respectively, and peaks in these extreme bins are considered experimental noise. |
| Replication | Sets of barcode controls were included, which contain the same sequence to test, but with different DNA barcodes, representing replicates. These replicates were performed in the context of the same bulk experiment. Examples are shown in Supplementary Figures 1E and 4C. |
| Randomization | This is not relevant as we are performing a bulk assay, therefore all variants are treated equally during data collection. |
| Blinding | This is not relevant as we are performing a bulk assay, therefore all variants are treated equally during data collection. |

# Reporting for specific materials, systems and methods

We require information from authors about some types of materials, experimental systems and methods used in many studies. Here, indicate whether each material, system or method listed is relevant to your study. If you are not sure if a list item applies to your research, read the appropriate section before selecting a response.

## Materials & experimental systems

| n/a | Involved in the study |
|---|---|
| ☒ | ☐ Antibodies |
| ☐ | ☒ Eukaryotic cell lines |
| ☒ | ☐ Palaeontology |
| ☒ | ☐ Animals and other organisms |
| ☒ | ☐ Human research participants |
| ☒ | ☐ Clinical data |

## Methods

| n/a | Involved in the study |
|---|---|
| ☒ | ☐ ChIP-seq |
| ☐ | ☒ Flow cytometry |
| ☒ | ☐ MRI-based neuroimaging |

## Eukaryotic cell lines

Policy information about cell lines

| | |
|---|---|
| Cell line source(s) | K562 cells (ATCC) |
| Authentication | No authentication |
| Mycoplasma contamination | The cells were tested negative for Mycoplasma. |
| Commonly misidentified lines (See ICLAC register) | None |

# Flow Cytometry

## Plots

Confirm that:

☒ The axis labels state the marker and fluorochrome used (e.g. CD4-FITC).

☒ The axis scales are clearly visible. Include numbers along axes only for bottom left plot of group (a 'group' is an analysis of identical markers).

☒ All plots are contour plots with outliers or pseudocolor plots.

☒ A numerical value for number of cells or percentage (with statistics) is provided.

## Methodology

| | |
|---|---|
| Sample preparation | K562 cells in culture are collected, washed in HBSS, filtered through a strainer and subjected to FACS analysis. |
| Instrument | FACS ARIA SORP |
| Software | BD FACSDiva |
| Cell population abundance | Sorted populations constituted typically 1-10% of the entire population. |
| Gating strategy | The gates for red and green fluorescence are shown in Supplementary Figure 1.<br>Sorting was performed with BD FACSAria II SORP (special-order research product) at low sample flow rate and a sorting speed of ~18000 cells/s. To sort cells that integrated the reporter construct successfully and in a single copy (~4% of the population), we determined a gate according to mCherry fluorescence so that only mCherry-expressing cells corresponding to a single copy of the construct were sorted (mCherry single population). We collected around 350 cells/variant on average for each library in order to ensure adequate library representation. Cells sorted for single integration of the transgene were grown for a week before we sorted the population into 16 (in the case of the stop-vector 12) bins according to GFP fluorescence, after gating for a narrow range of mCherry expression to avoid effects coming from the influence of the variable region on overall expression level. |

☒ Tick this box to confirm that a figure exemplifying the gating strategy is provided in the Supplementary Information.
