# Supplementary material for: High-throughput interrogation of programmed ribosomal frameshifting in human cells
Source: Nat Commun. 2020 Jun 16;11:3061. doi: 10.1038/s41467-020-16961-8 (PMC7297798; doi:10.1038/s41467-020-16961-8)
Supplement: Supplementary file 4 — Description of Additional Supplementary Files [file 41467_2020_16961_MOESM4_ESM.pdf]

Description of Additional Supplementary Files

File Name: Supplementary Data 1
Description: Previously reported PRF events tested here.

File Name: Supplementary Data 2
Description: Sequence, design information and experimental readouts for all library variants tested.

File Name: Supplementary Data 3
Description: Predicted secondary structure (pKiss) for library variants with systematic sequence alterations in the region downstream of the slippery site.
